# Clinical Outcomes of Myocarditis after Moderate-Dose Steroid Therapy in Systemic Sclerosis: A Pilot Study

**DOI:** 10.1155/2020/8884442

**Published:** 2020-12-19

**Authors:** Burabha Pussadhamma, Thapanee Tipparot, Naruemol Chaosuwannakit, Ajanee Mahakkanukrauh, Siraphop Suwannaroj, Ratanavadee Nanagara, Chingching Foocharoen

**Affiliations:** ^1^Department of Medicine, Faculty of Medicine, Khon Kaen University, Khon Kaen 40002, Thailand; ^2^Department of Radiology, Faculty of Medicine, Khon Kaen University, Khon Kaen 40002, Thailand

## Abstract

**Background:**

Myocarditis is reported in systemic sclerosis (SSc); however, treatment options and outcomes are limited. Our objective was to define cardiac outcomes after moderate-dose steroid therapy in SSc patients with myocarditis.

**Method:**

An open-label study was conducted among SSc patients with myocarditis—as defined by cardiovascular magnetic resonance (CMR), disease onset <5 years, and a NYHA functional class ≥II. All enrolled patients received prednisolone (30 mg/d) which would be tapered off by week 24, and CMR was followed up at the end of treatment.

**Results:**

A total of 20 SSc patients were enrolled which 12 patients completed the study. At week 24, 8 of the 12 cases experienced improvement of myocarditis. Compared to those with no improvement, these 8 patients had significantly longer disease duration (*p* = 0.03), higher heart rate at baseline (*p* = 0.049) and week 24 (*p* = 0.04), lower left ventricular (LV) and right ventricular (RV) stroke volume at baseline (*p* = 0.002 and *p* = 0.01) and week 24 (*p* = 0.01 and *p* = 0.02), and lower LV and RV cardiac output at week 24 (*p* = 0.01 and *p* = 0.01). Four cases died during follow-up (3 due to cardiac complications, 1 due to renal crisis). The two who died from heart failure had very high NT-prohormone-brain natriuretic peptide (NT-proBNP) and impaired LV ejection fraction (LVEF), and the one who died from arrhythmia had very high sensitivity of cardiac Troponin-T (hs-cTnT).

**Conclusions:**

Moderate-dose steroid therapy may improve myocarditis in SSc. A proportion of patients died due to cardiac complications during treatment, particularly those with high hs-cTnT, high NT-proBNP, and impaired LVEF. This trial is registered with NCT03607071.

## 1. Introduction

The hallmarks of systemic sclerosis (SSc) are microvascular disorders, autoimmune disturbances, and fibrosis of the skin and internal organs [1]. Since an effective treatment for scleroderma renal crisis was established, pulmonary arterial hypertension, interstitial lung disease, and cardiac involvement have overtaken as the leading causes of death in SSc [2, 3]. According to the European Scleroderma Trials and Research Group database, 26% of SSc-related death was defined as cardiac causes [3].

Intrinsic myocardial disease constitutes the majority of primary cardiac involvements in SSc [4–6]. The initial putative mechanism of myocardial disease in SSc is cardiac Raynaud's phenomenon for which inducible myocardial perfusion defects have been demonstrated through functional imaging studies [7–11], and associations between cardiac Raynaud's phenomenon and left ventricular systolic dysfunction, cardiac disease, or death were revealed in long-term follow-up studies [12, 13]. Functional (reversible) microvascular disorder can lead to structural (permanent) microvascular occlusion [14], and in turn to focal myocardial ischemia then myocardial fibrosis. Such pathological sequelae contribute to ventricular dysfunction, ranging from asymptomatic diastolic or systolic dysfunction, and clinically overt heart failure [6, 12].

Myocarditis has been secondarily raised as a potential disorder contributing to myocardial involvement in SSc. The coexistence between skeletal myositis and heart failure in patients with SSc suggests the possibility of myocarditis as an etiology for left ventricular dysfunction [15, 16]. Evidence of myocarditis in SSc is diagnosed noninvasively by echocardiography [16, 17] or even definitively by endomyocardial biopsy [18–20]. Cardiovascular magnetic resonance (CMR) has emerged as the best noninvasive diagnostic tool for myocarditis in SSc: with good accuracy and reproducibility [21]. CMR can differentiate acute versus chronic myocarditis in SSc [20] and can reveal myocarditis or myocardial fibrosis in SSc patients without cardiac symptoms [22, 23].

To date, standard treatment for myocarditis in SSc has not been established. Herein, we describe the outcome of moderate-dose steroid therapy in SSc patients with myocarditis diagnosed by CMR.

## 2. Methods

A prospective open-label study was conducted among 30 Thai adult SSc patients. The study included 2 phases. Phase 1 was the investigational phase. The patients who (a) had disease onset ≤5 years based on non-Raynaud's phenomenon of SSc symptoms; (b) had a NYHA functional class ≥ II; and (c) were followed-up at the Scleroderma Clinic, Khon Kaen University, between June 2018 and June 2019 underwent CMR and other laboratory tests on the same date. We excluded patients (a) taking steroids or immunosuppressants, having (b) a heart disease before being diagnosed with SSc, (c) life-threatening internal organ involvement in SSc that needs urgent high dose steroid or immunosuppressant therapy, (d) chronic renal failure (glomerular filtration rate <30 ml/min), (e) history of Gadolinium allergy, (f) metallic implant, (g) claustrophobia, (h) advanced stage of cancer, (i) being pregnant or lactation, (j) ongoing or current infection that needs systemic antibiotic therapy, (k) infection with viral hepatitis B or C, and (l) uncontrolled diabetes mellitus. The patients who had cardiac MRI findings compatible with myocarditis were given treatment in the phase 2 study. All medical treatment for their underlying SSc and concomitant medication apart from prohibit medication can be adjusted by a physician.

CMR was performed using STIR T2-weighted and T1-weighted before injection of 0.1 mmol/kg Gd-DTPA. Measurements were performed 1 minute after injection of Gd-DTPA for early gadolinium enhancement in the same area as in T2-weighted. Immediately after this, 0.1 mmol/kg of Gd-DTPA was given and late gadolinium-enhanced images were taken 15 min later.

**Figure 1:**
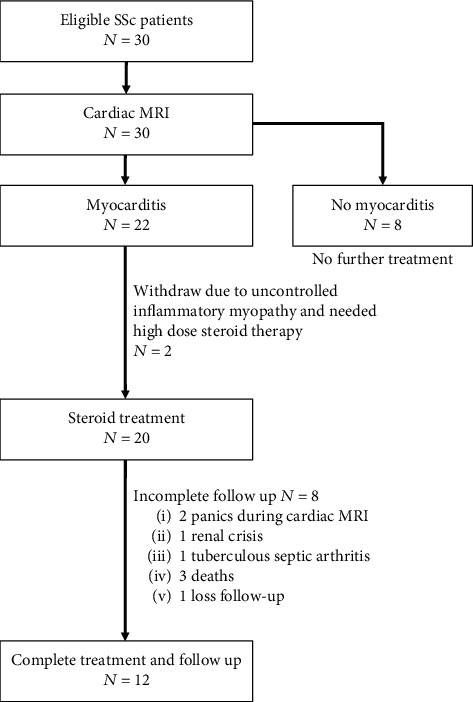
Flow diagram of the study.

Twenty-two SSc patients defined as having myocarditis by CMR received moderate-dose steroid treatment in a dose equivalent to prednisolone 0.5 mg/kg/d for 2 weeks then tapered off 10 mg every 2 weeks until the dose was 10 mg/d at week 4, then slowly tapered off completely by week 24 of treatment. The outcomes of myocarditis were assessed by CMR at week 24 after the start of treatment. A flowchart of the study is presented in Figure 1.

### 2.1. Study Endpoint

The study endpoint was assessed before starting treatment and at week 24 after treatment. The assessment included medical history, demographic data, SSc clinical characteristics, laboratory results, and CMR findings. SSc clinical characteristics, laboratory results, concomitant medications, and adverse events were evaluated and monitored at week 4, week 8, week 12, and week 24 during follow-up. A serious adverse event was reported if the adverse event led to hospitalization; there was new onset of heart failure, renal crisis, or death.

Any patient was withdrawn from the study if they had (a) serious side-effects related to steroid therapy including diabetic emergency (including diabetic ketoacidosis and hyperosmolar hyperglycemic nonketotic syndrome), (b) heart failure that needs rescue therapy, or (c) uncontrolled or opportunistic infection.

### 2.2. Operation Definitions

Diagnosis of SSc is based on the American College of Rheumatology criteria and/or fulfillment of the classification criteria of systemic sclerosis by the ACR/EULAR 2013 [24]. SSc is classified as limited cutaneous SSc (lcSSc) or diffuse cutaneous SSc (dcSSc), according to LeRoy et al. [25]. Onset of SSc is defined as the time of the first any non-Raynaud's phenomenon SSc symptom. Functional class—defined by the New York Heart Association (NYHA)—was subdivided into 4 functional classes [26]. Disease duration was calculated as the interval between disease onset and the time at the last data collection.

Myocarditis was defined as an inflammatory disease of the myocardium according to the CMR Lake Louise criteria [27], including at least 2 of the following criteria:

1. Regional or global myocardial signal intensity increased in the T2-weighted images
2. Increased global myocardial early enhancement ratio between the myocardium and skeletal muscle in the gadolinium-enhanced T1-weighted images; and
3. At least 1 focal lesion with nonischemic regional distribution in the inversion-recovery-prepared, gadolinium-enhanced, T1-weighted images (delayed enhancement)

A CMR study is consistent with myocyte injury or scarring caused by myocarditis if the third criterion is present and left ventricular dysfunction or pericardial effusion is present, indicating additional supportive evidence of myocarditis. Improvement of myocarditis by CMR was defined as the degree of myocarditis decreased without any progression of myocardial fibrosis or scarring when compared to the CMR baseline.

A high creatine kinase-MB (CK-MB) and a high erythrocyte sedimentation rate (ESR) was the serum CK-MB and the ESR level rose above 25 U/L and 25 mm/h, respectively. A high-sensitivity C-reactive protein (hsCRP) and high sensitivity of cardiac Troponin-T (hs-cTnT) was set at >5.0 mg/dl and >0.014 ng/ml. The latter exceeded the reference limit for the 99^th^ percentile [28, 29].

The definition of a prolonged QT interval was met at >440 ms in men and 460 ms in women [30]. The elevated cardiac enzyme was met when the hs-cTnT and/or CK-MB was elevated. The cut-off for the NT-prohormone-brain natriuretic peptide (NT-proBNP) level was by age group: a high NT-proBNP was defined as >450 pg/ml in persons under 50, >900 pg/ml in those between 50 and 75 and >1800 pg/ml in those over 75 [31].

Pulmonary involvement is defined by findings of pulmonary fibrosis, ground-glass opacity, or bronchiectasis performed by high-resolution, computed tomography (HRCT) of the chest. Pulmonary arterial hypertension is diagnosed when the mean pulmonary arterial pressure is >20 mmHg at rest with a pulmonary artery wedge pressure of ≤15 mmHg with a pulmonary vascular resistance of ≥3 Wood units, as confirmed by right heart catheterization [32]. The definition of anemia is fulfilled if hemoglobin is <12.0 g/dL in females and <13.0 g/dL in males [33]. Infection is defined as an adverse event if the patient needs systemic antibiotic treatment.

### 2.3. Machine Information

1.5 T CMR scanner is a product of SIEMENS AG 2012 Co., Ltd. Series number 522066 is licensed to Srinagarind Hospital, Thailand. Transthoracic echocardiography was performed by a cardiologist (BP) using the AlokaProSound F75 sonographic system (Hitachi-Aloka Medical, Ltd, Tokyo, Japan).

### 2.4. Statistical Analysis

Patient baseline characteristics were summarized using descriptive statistics: frequency (percentage) for categorical data, and mean (standard deviation) for continuous data. All of the patients were evaluated at week 24. The clinical characteristics were compared between baseline and week 24 using the Wilcoxon test for continuous data and chi-square or Fisher's Exact Test as appropriate for the categorical data. A *p* value < 0.05 was considered statistically significant. All analyses were done using STATA version 16.0 (StataCorp., College Station, TX, USA).

### 2.5. Ethical Consideration

The study protocol was reviewed and approved by the Ethics Committee of Khon Kaen University for human Research based on the Declaration of Helsinki and the ICH Good Clinical Practice Guidelines (HE611134). All participants signed informed consent before enrolling. The sponsor had no role in the study.

## 3. Results

Of the 30 SSc patients with NYHA functional class II who underwent CMR, 22 (73.3%) had myocarditis, and 20 (66.7%) were given steroid treatment as per protocol (2 were withdrawn from the study because of uncontrolled inflammatory myopathy and needed high-dose steroid and immunosuppressive therapy). Of those who received steroid treatment, the majority had the dcSSc subset (15 cases; 75%) and were female (13 cases: 65%). Most of the patients (19 cases; 95%) were positive for anti-topoisomerase I antibody, and the remainder were positive for the anti-centromere antibody. The clinical characteristics at baseline are presented in Table 1.

Eight patients were not able to complete the study; 2 panicked during the CMR (at week 24), 3 died due to cardiac complications (2 to heart failure and 1 to moderate pericardial effusion with cardiac arrhythmia), 1 died due to a scleroderma renal crisis, 1 left the study because of having tuberculous septic arthritis, and 1 was lost to follow-up (Figure 1).

**Figure 2:**
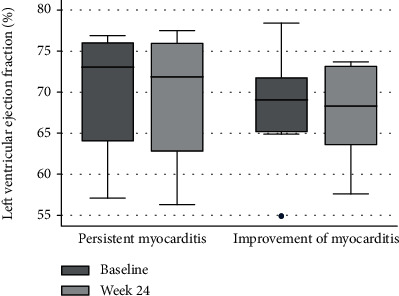
Left ventricular ejection fraction by cardiovascular magnetic resonance comparison between baseline and week 24 after steroid treatment classified by response to treatment.

At week 24 after treatment, 8 of the 12 cases (66.7%) experienced improvement of their myocarditis by CMR. The patients who had improvement of their myocarditis after steroid treatment—over against those who had persistent myocarditis—had a significantly longer duration of disease, a higher heart rate at baseline and week 24, a higher left ventricular end-diastolic volume at baseline and week 24, a lower left ventricular stroke volume at baseline and week 24, a lower right ventricular end-diastolic volume, a right ventricular end-systolic volume and right ventricular cardiac output at week 24, and a lower right ventricular stroke volume at baseline and week 24 (Table 2). The clinical characteristics at baseline and week 24 after treatment—between those who had improvement of myocarditis and those with persistent myocarditis—are presented in Table 2. When compare left ventricular ejection fraction by CMR between baseline and week 24 after steroid treatment, there was no significant difference of left ventricular ejection between baseline and after steroid treatment in both the patients with persistent myocarditis and with improvement of myocarditis (Figure 2).

Thirty-six adverse events were reported (Table 3), of which infection was the most common (viz., urinary tract infection 8 events and upper respiratory tract infection 2 events) and most (16 events) were reported at week 4 after starting steroid treatment. Four patients died during follow-up. All the patients had dcSSc and were positive for the anti-topoisomerase antibody, tendon friction rub, high NT-proBNP, and pericardial effusion. Details of the clinical characteristics and baseline CMR findings of the cases who died are presented in Table 4.

## 4. Discussion

The prognosis of SSc patients with active myocarditis and overt heart failure is poor, and treatment with immunosuppressive drugs yields only an uncertain result. West et al. reported the late occurrence of a life-threatening, conduction system defect in SSc patients with myocarditis while on steroid therapy [15]. Clemson et al. reported a patient with myocarditis and heart failure at early onset of SSc; the patient received treatment with methylprednisolone but died shortly thereafter [18]. Kerr et al. reported death due to heart failure in SSc patients with myocarditis treated with steroid alone [16]. Carette et al., however, describe a patient with SSc and severe myocarditis who responded to intravenous pulse methylprednisolone [17]. Successful control of myocarditis in SSc is reported using a combination of azathioprine and prednisolone [19] or cyclophosphamide and methylprednisolone [34]. Pieroni et al. describe a promising response in 7 SSc patients with symptomatic myocarditis as symptoms and cardiac enzymes improve with immunosuppressive therapy, albeit sudden death occurred after treatment in 2 patients [20]. Recently, Mavrogeni et al. describe the recovery of myocarditis in SSc patients with silent myocarditis treated with prednisolone and azathioprine [23].

We first described the outcomes of moderate-dose steroid therapy alone in mildly symptomatic SSc patients with myocarditis in a prospective fashion. The prevalence of CMR-diagnosed myocarditis in symptomatic SSc patients was high. Most of the patients had normal left ventricular ejection fraction, indicating that we had enrolled patients at an early phase of myocarditis. We also found that among all the SSc patients who had myocarditis, most had high levels of hs-cTnT, while a few had high levels of NT-proBNP, and only a few had impaired left ventricular systolic function. Some died due to cardiac causes shortly after diagnosis, highlighting the prevalence of myocarditis among symptomatic SSc patients and emphasizing its poor prognosis.

The majority of the studied population **(**80%) responded positively or were stabilized with steroid therapy. The outcomes of moderate-dose steroid therapy in our study could be classified into 3 categories according to the cardiac response: (a) improved myocarditis, (b) persistent myocarditis with no cardiac event, and (c) treatment failure with cardiac event. Most of the patients were classified as having improved myocarditis, which is significant because this group typically experienced longer disease duration, higher heart rate, and lower stroke volume at baseline and 24 weeks compared with the group with persistent myocarditis. The underlying mechanism is unknown for why disease duration affects the response of steroid therapy. It is possible that the etiology of myocarditis is different between early and late-onset SSc. A recent study of 7 SSc patients with myocarditis, however, showed no obvious differences from endomyocardial biopsy vis-à-vis histology and immunohistochemistry between those who had early vs. late onset myocarditis in SSc [20]. A larger data set of endomyocardial biopsy from SSc patients with myocarditis at various times of disease onset is required to clarify this issue.

In terms of hemodynamics, lower stroke volume was significantly found at baseline in patients with improved myocarditis. To the best of our knowledge, there has been no comprehensive hemodynamic study in patients with mildly symptomatic or early myocarditis with which we can compare. We postulate that this might be due to different etiology, say those with improved myocarditis being **“**true**”** myocarditis or a steroid-responsive type vs. those with persistent myocarditis being **“**false**”** myocarditis or a steroid-nonresponsive type. Lower stroke volume in patients with improved myocarditis might be a result of occult, early cardiac dysfunction, and high heart rate in these patients might be a logical compensatory response to low stroke volume or part of the primary cardiac involvement of SSc in the cardiac conduction system. Trends of slight elevation of ESR and CRP at baseline and a significant reduction of ESR after steroid therapy in the improved myocarditis group might support our hypothesis that inflammation preexisted and played role in its own pathogenesis. A significant reduction of CK-MB in patients with improved myocarditis after steroid therapy was also found and might indicate the coexistence of subclinical myositis. It is understood that myositis and myocarditis can be coincident in SSc patients [15, 16]; thus, this finding could support speculation of a **“**true**”** myocarditis in the improved myocarditis group. A trend to a slight elevation of hs-cTnT and NT-proBNP at baseline in patients with improved myocarditis might support the significant hemodynamic disturbance described above.

Although improvement of myocardial inflammation was found in the improved myocarditis group, the initial hemodynamic disturbances were not recovered, and significant lower cardiac output was detected at the end of the study. This indicated that cardiac dysfunction due to myocarditis in SSc might persist even after recovery of acute inflammation, or an initial active myocarditis could become noninflamed cardiomyopathy even after steroid therapy. Unchanged levels of hs-cTnT and NT-proBNP after steroid therapy in improved myocarditis group also corresponded with persistent hemodynamic impairment. Some patients were classified as having persistent myocarditis with no cardiac event, as their hemodynamic findings from transthoracic echocardiography and CMR, hs-cTnT levels, and NT-proBNP levels were unremarkable, and their inflammatory markers were not significantly changed. These findings might give some clues relating to the putative false myocarditis postulation.

Further systematic, large-scale, long-term study, complete with hemodynamic and tissue histopathologic study is needed to confirm our explanations of the hemodynamic consequences, cardiac biomarkers, and inflammatory markers—as related to steroid therapy response in SSc patients with myocarditis. Notwithstanding, according to our current study findings, we found that low stroke volume and high heart rate in SSc patients with myocarditis was suggestive of an expected improvement after steroid therapy. By contrast, left ventricular ejection fraction which is a more common indicator gave no such guide.

Three patients **(**15**%)** died from cardiac causes during the study. Unfortunately, the number of deaths was too low to perform a valid statistical analysis; however, we observed that among the patients who died from cardiac causes, all had dcSSc—the two who died from heart failure had very high NT-proBNP levels, slightly high hs-cTnT levels, and severely impaired left ventricular systolic function **(**left ventricular ejection fraction <35**%)**, and the one who died from cardiac arrhythmia had high normal NT-proBNP level but remarkably high hs-cTnT level though normal left ventricular ejection fraction. It could be roughly summarized that in mildly symptomatic SSc patients with myocarditis, the presence of either high NT-proBNP levels or high hs-cTnT levels or having impaired left ventricular systolic function are likely predictors of a grave outcome, and treatment with moderate-dose steroid alone would likely not remedy the condition. There are case reports of failure of steroid therapy alone in overt, severely symptomatic myocarditis in SSc patients [15, 16, 18], and our results just confirmed the ineffectiveness of steroid therapy alone in SSc patients with myocarditis with severe anatomical or functional cardiac disorders, even just mildly symptomatic. Perhaps an escalated and combined therapy with other immunosuppressive agents should be considered for such patients as there is evidence of success with those therapies in SSc patients with either silent [23] or overt, severe myocarditis [17, 19, 20, 34].

Antoniades et al. described an improvement of left ventricular ejection fraction after moderate-dose steroid therapy alone in SSc patients who initially had impaired left ventricular systolic function [35]. Importantly, the study was done in so-called asymptomatic patients, suggesting that moderate-dose steroid therapy alone might be sufficient for SSc patients with early myocarditis; supporting our results. NT-proBNP and hs-cTnT were not, however, assessed in the study by Antoniades et al., preventing generalization of interpretation and comparison of cardiac dysfunction between studies. Recently, treatment of myocarditis in SSc has been broadly recommended as immunosuppressive agents plus steroid therapy [36, 37]. Our results may provide some insight into the role of moderate-dose steroid therapy alone for myocarditis in SSc and add more information for building future treatment recommendations.

Despite moderate-dose steroid treatment for myocarditis being effective in around two-thirds of our SSc patients, the adverse events vs. benefits must be weighed. According to the major concern of risk—viz., infection and renal crisis—in SSc patients on ≥15 mg prednisolone/d [26], the clinical signs and symptoms of infection and glomerular filtration rate were closely monitored during steroid treatment. Infection was, nevertheless, the most common adverse event in our patients. In addition, one patient was removed from the study because of having tuberculous septic arthritis. Although the infection was not serious enough to lead to high morbidity in those patients and none had opportunistic infection, the infection rate increases in SSc patients on steroid treatment. As well as renal crisis, only one patient (5%) had renal crisis but the patient died. As a consequence, the risk and benefits of moderate-dose steroid treatment for myocarditis in SSc should be weighed.

Although the current study did not focus on screening myocarditis in SSc, our findings suggest that the use of hs-cTnT and CK-MB—albeit not NT-proBNP and basic echocardiographic parameters—are helpful in detecting early myocarditis in SSc patients with otherwise unexplained dyspnea. In this regard, further CMR seems to be the best way to confirm the diagnosis; however, one must consider the risk of contrast media, cost burden, and availability before using CMR. More data is needed to justify the algorithm of CMR referral for appropriate diagnosis of myocarditis in SSc.

The strength of our study is that it is the first prospective study evaluating outcomes of moderate-dose steroid therapy alone in a large number of mildly symptomatic SSc patients with myocarditis using CMR as the method of detailed cardiac evaluation. Limitations include the following: (a) we did not perform endomyocardial biopsy, so definite myocarditis could not be confirmed, nor did we perform autopsy of the patients who died, so we cannot prove whether active myocarditis was present at death; (b) myocardial tissue was not taken, so we are not able to rule out infective myocarditis where viral myocarditis is diagnosed in 3 of 7 SSc patients with myocarditis [20] so the response of infective myocarditis to moderate-dose steroid therapy cannot be concluded; (c) we did not combine immunosuppressive drugs in our regimen, even though a combination of immunosuppressive drugs and moderate-dose steroid might give a better result, as prednisolone and azathioprine have been reported to normalize myocarditis in SSc patients with subclinical myocarditis after 6 months therapy [23]; and (d) although we had enrolled quite a large number of patients for this rare condition, this number is too small to affirm a statistically valid and reliable conclusion.

## 5. Conclusions

The prevalence of myocarditis in mildly symptomatic SSc patients is high and early diagnosis of this condition could be guided by high levels of hs-cTnT and CK-MB. The possible outcomes of moderate-dose steroid therapy in SSc patients with myocarditis are (a) improved myocarditis, (b) persistent myocarditis, or (c) treatment failure. Treatment with moderate-dose steroid seems to be adequate in SSc patients with myocarditis with no obvious cardiac dysfunction, for which low stroke volume and high heart rate could be indicators of a promising response. The outcome of such therapy will not be good when applied to SSc patients who have significant cardiac dysfunction—i.e., significantly high levels of NT-proNBP or hs-cTnT, or impaired left ventricular systolic function. Further long-term research should be done to clarify the outcomes of moderate-dose steroid therapy in patients with this condition.

## Figures and Tables

**Table 1:** Overall baseline clinical characteristics.

Variable	*N* = 20
Female (%)	13 (65)
Age on enrollment (years); mean ± SD	52.8 ± 15.3
BMI (kg/m^2^); mean ± SD	21.0 ± 2.8
Duration of disease (years); mean ± SD	3.2 ± 1.3
Diffuse cutaneous SSc (%)	15 (75)
NYHA functional class II	20 (100)
Serology	
Anti-topoisomerase antibody positive (%)	19 (95)
Anti-centromere antibody positive (%)	1 (5)
Clinical characteristic	
Raynaud's phenomenon (%)	12 (60)
Digital ulcer (%)	3 (15)
Edematous skin (%)	11 (55)
Tendon friction rub (%)	12 (60)
Hand deformity (%)	14 (70)
Esophageal involvement (%)	4 (20)
Stomach involvement (%)	3 (15)
Modified Rodnan skin score (points); mean ± SD	13.6 ± 9.4
Pulmonary involvement (%)	12 (60)
Pulmonary arterial hypertension (%)	3 (15)
Renal crisis (%)	0
Laboratory result	
Anemia (%)	9 (45)
Hemoglobin (g/dL); mean ± SD	11.9 ± 1.7
Erythrocyte sedimentation rate > 25 mm/h (%)	17 (85)
C-reactive protein >5 mg/dl (%)	13 (65)
CK-MB >25 U/L (%)	12 (60)
Hs-cTnT >0.014 ng/ml (%)	16 (80)
Hs-cTnT; mean ± SD	183.9 ± 37.8
High NT-proBNP (%)	4 (20)
NT-proBNP; mean ± SD	777.8 ± 128.2
Estimated GFR (ml/min/1.73m^2^); mean ± SD	98.3 ± 23.7
Forced vital capacity (%predicted); mean ± SD	65.9 ± 11.1
Radiographic result	
Cardiomegaly by chest radiography (%)	5 (25)
Ground glass opacification by HRCT chest (%)	12 (60)
Echocardiography result	
LV ejection fraction (%)	59.4 ± 12.6
Impaired LV systolic function (LV ejection fraction <50%) (%)	2 (10)
Tricuspid velocity jet (m/sec)	2.3 ± 0.3
Right ventricular systolic pressure (mmHg); mean ± SD	26.7 ± 6.2
E' velocity (cm/sec); mean ± SD	7.2 ± 2.5
Pericardial effusion (%)	8 (40)
CMR finding	
LV ejection fraction (%); mean ± SD	63.7 ± 12.8
LV stroke volume (ml); mean ± SD	62 ± 25.1
LV cardiac output (L/min); mean ± SD	4.9 ± 1.4
RV ejection fraction (%); mean ± SD	54.3 ± 10
RV stroke volume (ml); mean ± SD	64.7 ± 20.2
RV cardiac output (L/min); mean ± SD	5.0 ± 1.4
Pericardial effusion (%)	16 (80)

BMI denotes body mass index; CK-MB: creatine kinase-muscle/brain; CMR: cardiovascular magnetic resonance; GFR: glomerular filtration rate; hs-cTnT: high sensitivity of cardiac Troponin-T; HRCT: high-resolution computed tomography; LV: left ventricular; NT-proBNP: NT-prohormone-brain natriuretic peptide; NYHA: New York Heart Association; RV: right ventricular; SD: standard deviation; SSc: systemic sclerosis.

**Table 2:** Clinical characteristics at baseline and week 24 after treatment in those who had improvement of myocarditis vs. persistent myocarditis.

Variable	Persistent myocarditis *N* = 4		*p* value	Improvement of myocarditis *N* = 8		*p* value	*p* value^1^	*p* value^2^
	Baseline	Week 24		Baseline	Week 24			
Female (%)	2 (50)		—	8 (100)		—	0.09	—
Age on enrollment (years); mean ± SD	54.3 ± 10.8	—		51.6 ± 23.1			0.83	
Duration of disease (years); mean ± SD	2.1 ± 0.4	—		4.0 ± 1.4			0.03^∗^	
Diffuse cutaneous SSc (%)	3 (75)		—	5 (62.5)		—	0.99	—
NYHA functional class at the end of study							0.99	
I	1 (25)			2 (50)				
II	3 (75)			5 (62.5)				
III	0			1 (12.5)				
Serology								
Anti-topoisomerase antibody positive (%)	4 (100)			7 (87.5)		—	0.99	—
Anti-centromere antibody positive (%)	0		—	1 (12.5)		—	0.99	—
Clinical characteristic								
Heart rate	78.5 ± 14.3	78.2 ± 10.2	0.96	99.6 ± 12.7	93.6 ± 10.4	0.15	0.049^∗^	0.042^∗^
Raynaud's phenomenon (%)	3 (75)	1 (25)	0.15	4 (50)	1 (12.5)	0.38	0.58	0.99
Digital ulcer (%)	1 (25)	0	0.99	2 (25)	1 (12.5)	0.99	0.99	0.99
Edematous skin (%)	4 (100)	0	0.04^∗^	3 (37.5)	0	0.15	0.08	NA
Tendon friction rub (%)	2 (50)	1 (25)	0.99	2 (25)	1 (12.5)	0.99	0.55	0.99
Hand deformity (%)	2 (50)	3 (75)	0.99	5 (62.5)	4 (50)	0.99	0.99	0.99
Esophageal involvement (%)	0	0	NA	1 (12.5)	2 (25)	0.99	0.99	0.49
Stomach involvement (%)	1 (25)	0	0.99	2 (25)	1 (12.5)	0.99	0.99	0.99
Modified Rodnan skin score (points); mean ± SD	10.3 ± 4.9	16.8 ± 12.2	0.18	11.3 ± 10.8	11.3 ± 12.9	0.64	0.86	0.51
Pulmonary arterial hypertension (%)	1 (25)	1 (25)	0.99	1 (12.5)	1 (12.5)	0.99	0.99	0.99
Renal crisis (%)	0	0	NA	0	0	NA	NA	NA
Laboratory result								
Erythrocyte sedimentation rate (mm/hr)	44.5 ± 32.9	49.6 ± 41.2	0.76	57.6 ± 29.3	43.9 ± 25.8	0.01^∗^	0.50	0.79
C-reactive protein (mg/dl)	2.9 ± 2.6	4.3 ± 2.3	0.08	7.3 ± 7.2	5.6 ± 7.2	0.22	0.29	0.74
CK-MB (U/L)	27.5 ± 9.6	23 ± 7.3	0.49	34.8 ± 20.3	17.9 ± 4.6	0.04^∗^	0.52	0.18
hs-cTnT (pg/ml)	41 ± 20.1	56.2 ± 41.3	0.40	89 ± 34.7	68.6 ± 49.0	0.60	0.52	0.86
NT-proBNP (pg/ml)	78 ± 54.7	99 ± 37	0.92	152.6 ± 108.1	182 ± 101.7	0.80	0.23	0.51
Radiographic result								
Cardiomegaly by chest radiography	3 (75)	0	0.08	3 (42.9)	1 (12.5)	0.32	0.55	0.38
Ground glass opacification by HRCT	3 (75)	—	—	4 (50)	—	—	0.58	—
Echocardiography result								
LV ejection fraction (%)	60.1 ± 7.6			66.2 ± 7.8			0.23	—
Tricuspid velocity jet (m/sec)	2.37 ± 0.39			2.34 ± 0.31			0.87	—
Right ventricular systolic pressure (mmHg); mean ± SD	28.4 ± 8.8			26.9 ± 6.9			0.75	
CMR finding								
LV ejection fraction (%); mean ± SD	70.0 ± 9	69.4 ± 9.4	0.26	68.2 ± 6.9	67.7 ± 5.9	0.85	0.70	0.70
LV stroke volume (ml); mean ± SD	90.5 ± 19.6	76.7 ± 11.3	0.32	56 ± 9.7	57.0 ± 10.6	0.17	0.002^∗^	0.01^∗^
LV cardiac output (L/min); mean ± SD	5.4 ± 1.2	5.7 ± 0.6	0.44	4.6 ± 0.9	4.2 ± 0.8	0.18	0.20	0.01^∗^
LV mass (g); mean ± SD	89 ± 29.8	87.8 ± 14.1	0.90	70.9 ± 13.6	73.6 ± 17.7	0.56	0.17	0.19
LV mass index (g/m^2^); mean ± SD	55.7 ± 12.8	55.6 ± 3.5	0.99	48.9 ± 8.6	51.1 ± 13.3	0.52	0.30	0.53
RV ejection fraction (%); mean ± SD	58.8 ± 4.6	57.9 ± 8.1	0.74	57.0 ± 8.1	59 ± 2.4	0.46	0.71	0.73
RV stroke volume (ml); mean ± SD	80.3 ± 16.3	76.1 ± 18.2	0.25	54.0 ± 11.5	54.5 ± 8.7	0.83	0.01^∗^	0.02^∗^
RV cardiac output (L/min); mean ± SD	5.3 ± 1.1	5.6 ± 1.1	0.57	4.4 ± 0.9	4.0 ± 0.7	0.12	0.14	0.01^∗^
Pericardial effusion	2 (50)	2 (50)	0.99	7 (87.5)	5 (62.5)	0.50	0.24	0.99

^1^Comparison of clinical characteristics at baseline between those with SSc who had improvement of myocarditis vs. persistent myocarditis. ^2^Comparison of clinical characteristics at week 24 between those with SSc who had improvement of myocarditis vs. persistent myocarditis. CK-MB denotes creatine kinase-muscle/brain; CMR: cardiovascular magnetic resonance; hs-cTnT: high sensitivity of cardiac Troponin-T; HRCT: high-resolution computed tomography; LV: left ventricular; NT-proBNP: NT-prohormone-brain natriuretic peptide; NYHA: New York Heart Association; RV: right ventricular; SD: standard deviation; SSc: systemic sclerosis.

**Table 3:** Adverse events.

Adverse event	Number of events (%)
Total event	36 (100.0)
Infection	10 (27.8)
Gastroesophageal reflux disease	4 (11.1)
Dyslipidemia	3 (8.3)
Gastrointestinal discomfort	3 (8.3)
Digital ulcer	3 (8.3)
Uncontrolled diabetes mellitus	2 (5.6)
Heart failure	2 (5.6)
Cardiac arrhythmia	1 (2.8)
Fatigue	1 (2.8)
Insomnia	1 (2.8)
Scleroderma renal crisis	1 (2.8)
Neuropathy	1 (2.8)
Other	4 (11.1)

**Table 4:** Clinical data of patients who died.

Variable	Case 1	Case 2	Case 3	Case 4
Sex	Female	Male	Female	Male
Age on enrollment (years)	60.2	55.1	56.4	58
BMI (kg/m^2^)	17.2	18.6	19.6	19.1
Duration of disease (years)	4.5	2.8	1.7	4
SSc subset	dcSSc	dcSSc	dcSSc	dcSSc
Anti-topoisomerase antibody positive	Positive	Positive	Positive	Positive
Clinical characteristics on an enrollment				
Tendon friction rub	Yes	Yes	Yes	Yes
Modified Rodnan skin score (points)	17	21	26	33
Pulmonary involvement	Yes	Yes	Yes	Yes
Pulmonary arterial hypertension	No	Yes	No	No
Laboratory results on enrollment				
Erythrocyte sedimentation rate (mm/hr)	61	47	99	65
C-reactive protein (mg/dl)	11.3	21.8	46.4	44.7
CK-MB (U/L)	17	27	161	70
hs-cTnT (pg/ml)	42.1	181.4	1704	403.7
NT-proBNP (pg/ml)	4128	3306	862	2410
Estimated GFR (ml/min/1.73m^2^); mean±SD	95.8	98.6	125.7	94.5
Cardiomegaly by chest radiography	Yes	No	No	No
Ground glass opacification by HRCT chest	Yes (<20%)	Yes (<20%)	No	Yes (<20%)
Echocardiography results on enrollment				
LV Ejection fraction (%)	26.1	33.5	68.0	53.5
Tricuspid velocity jet (ms)	1.83	2.5	2.1	2.3
Right ventricular systolic pressure (mmHg)	23.4	35	20	23.4
E' velocity (cm/sec)	NA	NA	NA	4.8
Pericardial effusion	Trivial	No	No	No
CMR findings on enrollment				
LV ejection fraction (%)	30.3	31.8	66.3	64.9
LV stroke volume (ml)	32.9	99.5	53.9	49.5
LV cardiac output (L/min)	2.3	4.6	6.4	3.5
LV mass (g)	100	120.5	68.5	115.8
LV mass index (g/m^2^)	81.6	74.3	49.4	78.5
RV ejection fraction (%)	30	39.2	64.7	44.6
RV stroke volume (ml)	56.4	99.5	57	47.1
RV cardiac output (L/min)	3.95	6.9	6.7	3.3
Pericardial effusion	Moderate	Minimal	Minimal	Minimal
Duration of death after enrollment (months)	3.8	1.1	2	3.7
Cause of death	Heart failure	Heart failure	Cardiac arrhythmia (VT)	Renal crisis

BMI denotes body mass index, CK-MB; creatine kinase-muscle/brain, CMR; cardiovascular magnetic resonance, dcSSc; diffuse cutaneous systemic sclerosis, GFR; glomerular filtration rate, hs-cTnT; high sensitivity of cardiac Troponin-T, HRCT; high resolution computed tomography, LV; left ventricular, NT-proBNP; NT-prohormone-brain natriuretic peptide, RV; right ventricular, SSc; systemic sclerosis, VT; ventricular tachycardia.

## Data Availability

Data or materials are available upon request.
